# Evidence‐based practice in radiography: A strategy for shifting our culture

**DOI:** 10.1002/jmrs.801

**Published:** 2024-06-04

**Authors:** Laura Di Michele, Amani Bell, Kate Thomson, Warren Reed

**Affiliations:** ^1^ Faculty of Medicine and Health, Sydney School of Health Science University of Sydney Camperdown New South Wales Australia

## Abstract

Evidence‐based practice (EBP) has a vital role to play in improving outcomes for patients, organisations and individual practitioners. Unfortunately, within diagnostic radiography, literature consistently demonstrates that positive EBP is not the norm. This editorial discusses a strategy for fostering cultural change within the profession to improve EBP.

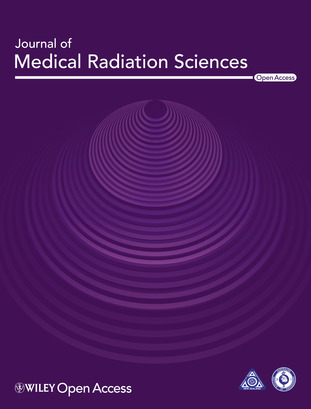

Evidence‐based practice (EBP) requires practitioners to integrate four primary pillars: clinical expertise, research evidence, patient preferences and pragmatics and context. Due to its nature EBP is challenging, as practitioners must flexibly adapt their knowledge to individual clinical situations to provide the best outcomes for their patients. Evidence‐based practice has significant impacts on three key groups; it improves outcomes for patients, increases efficiency for organisations and enhances job satisfaction for individual practitioners.^1^, ^2^

To facilitate evidence‐based practice, practitioners must possess a diverse skillset that extends beyond technical radiography skills, to encompass skills across the EBP cycle. While training and education in EBP are essential for initial skill development and confidence, formal university‐based education can only provide a foundation. Beyond skill acquisition, the application of EBP demands that practitioners commit to lifelong learning and prioritise meaningful continuous professional development. Unfortunately, allied health graduates often experience a loss of confidence in their EBP skills within 5 years of graduation.^3^ The current approach to practice is ineffective in translating evidence‐based practices within the field of diagnostic radiography. Why is this, and what actions can we take as a discipline to address it?

Literature from other disciplines suggests that an individual's beliefs and attitudes about EBP significantly influence their likelihood of practicing in an evidence‐based manner. Interestingly, the field of diagnostic radiography appears to differ on this point. In this issue, Melesse, Amde and Tezera^4^ provide an overview of EBP in Addis Ababa, Ethiopia. The results of their paper are consistent with the results of our recent survey of Australian radiographers^5^ and a systematic review of studies of radiographers internationally.^6^ While individual radiographers generally hold very positive attitudes towards EBP, the application of this practice is limited and variable.

Workplace culture plays a crucial role in the implementation of EBP, both in terms of prioritisation and socialisation.^2^, ^7^ A positive EBP culture within organisations affects individuals' knowledge, beliefs, competency and the implementation of evidence‐based practices. Moreover, it is positively associated with higher job satisfaction and intention to stay.^7^ Unfortunately, within the field of diagnostic radiography, the literature consistently demonstrates that strong, positive research cultures and EBP cultures are not the norm.^6^ Therefore, there is a pressing need to invest at a professional, organisational and individual level to foster a culture conducive to EBP implementation.

To address the lack of application of EBP in diagnostic radiography, we need coordinated, strategic efforts to improve our professional culture. Radiographers' traditional role has been as consumers, rather than creators of empirical evidence,^8^ with few displaying interest, confidence or experience in undertaking research.^9^, ^10^ Despite an increase in higher level degrees within the profession, very few Australian radiographers undertake formal research training.^10^ At a professional level, we must reclaim our knowledge base, by providing targeted funding for radiographers to engage in both research and other EBP activities. To ensure this research is relevant and patient centred, it is crucial to involve patients in the research process. Patient engagement not only enhances the relevance of interventions and cultural sensitivity but also has benefits for both patients and researchers alike.^11^

To enable this, we must ensure that practitioners are enabled to undertake further studies and research training. A 2022 survey of Australian radiographers showed that only 1.6% of respondents with a medical imaging background had a PhD, which was much lower than the 9.8% among radiation therapists.^10^ Efforts must be made to recruit and train talented diagnostic radiographers to engage with high quality research. Moreover, there must be well‐defined pathways for career progression and advancement, ensuring the discipline retains high achieving, experienced, evidence‐based professionals who are committed to evidence‐based practices within the discipline.

A recent scoping review investigating the outcomes of EBP initiatives found that 94% of articles reporting on return on investment showed a positive return, with none reporting negative returns.^1^ Positive research and EBP cultures are associated with higher organisational performance, productivity and staff satisfaction and retention.^12^ Strong EBP culture builds strong organisations; thus, organisations cannot afford to neglect prioritising and investing in EBP.

At an organisational level, it is crucial to visibly prioritise EBP. Examples of such visibility include integrating EBP in organisational values, job descriptions and key performance indicators. Given that individuals with higher degrees are more likely to maintain EBP confidence,^3^ prioritising recruitment of individuals with higher degrees may help improve the culture within individual organisations. Additionally, supporting current employees to upskill and undertake further studies will also help organisations in enhancing their EBP practices. It is vital that translational skills are embedded in the curriculum of advanced degrees. This ensures that graduates possess the skills to apply the knowledge gained during their studies within their individual settings, thereby fostering evidence‐based change within their departments.

The paper by Melesse, Made and Tezera R^4^ in this issue, along with our Australian study^5^ and an international literature review^6^ all highlight that radiographers struggle to find the time to prioritise EBP. Managers must ensure that every radiographer has dedicated time set aside to engage in EBP activities within their workday and provide adequate channels for dissemination among colleagues. Furthermore, time to actively apply a patient centred, EBP approach to medical imaging must be built into scheduling, ensuring that patient preferences are heard and prioritised.

Individual radiographers also have a role to play. Senior radiographers can act as EBP champions within their organisations. They can actively contribute to the cultural shift and can do this by upskilling, role modelling and acting as mentors to the next generation of radiographers.^7^ EBP mentorship has been shown to have strong positive impact on implementation.^7^ This mentorship by senior radiographers could assist in the development of the clinical expertise pillar of EBP, helping junior radiographers to develop skills and find the intricate balance between the often‐competing pillars of EBP. Likewise, as mentees, junior radiographers can bring their expertise in individual EBP skills to the table, sharing and utilising the EBP skills that they have graduated with more recently from university. By actively employing and refining these skills in the clinical environment, these clinicians are less likely to display the loss of confidence that has been previously reported in other disciplines.^3^

In conclusion, with solid international baselines as our guide,^4^, ^5^, ^6^ it is imperative to implement a resolute strategy for advancing EBP within diagnostic radiography. This entails fostering a pervasive culture of EBP across all levels – professional, organisational and individual. By driving this transformation, we can not only enhance patient outcomes and organisational success but also elevate staff satisfaction and retention. Creating this cultural shift and enhancing the implementation of EBP within the profession ensures a legacy of excellence for generations of radiographers to come.

## Conflict of Interest

The authors declare no conflict of interest.

## Data Availability

Data sharing not applicable to this article as no datasets were generated or analysed.
